# Evaluation of the Relationship Between Salivary Adipokine Levels With Appetite and Anthropometric Indices in Patients With Type 2 Diabetes

**DOI:** 10.1002/edm2.70012

**Published:** 2024-11-13

**Authors:** Amir Reza Akbarzadeh, Shiva Borzouei, Salman Khazaei, Mina Jazaeri

**Affiliations:** ^1^ Department of Oral and Maxillofacial Medicine, Dental Research Center, School of Dentistry Hamadan University of Medical Sciences Hamadan Iran; ^2^ Endocrinology & Metabolism, Department of Internal Medicine, School of Medicine Hamadan University of Medical Sciences Hamadan Iran; ^3^ Epidemiology, Department of Epidemiology, School of Health Hamadan University of Medical Sciences Hamadan Iran; ^4^ Oral & Maxillofacial Medicine, Department of Oral and Maxillofacial Medicine, Dental Research Center, School of Dentistry Hamadan University of Medical Sciences Hamadan Iran

**Keywords:** adipokine, anthropometric indices, appetite, chemerin, interleukins, leptin, resistin

## Abstract

**Objective:**

This study aimed to evaluate the association between salivary adipokine levels, including leptin, chemerin, resistin and interleukin‐6, with body mass index (BMI), waist and wrist circumference and appetite in patients with type 2 diabetes.

**Methods:**

In this cross‐sectional study, 104 participants were divided into three groups: 35 diabetic patients, 35 pre‐diabetic individuals and 34 healthy controls. Unstimulated saliva samples were collected using the spitting method, and salivary levels of leptin, chemerin, resistin and interleukin‐6 were measured via ELISA. Appetite was assessed using a standard questionnaire, and BMI, waist and wrist circumferences were measured with a tape measure. Statistical analysis was performed using SPSS 26, with a significance threshold set at 0.01.

**Results:**

Significant differences were found in the salivary levels of leptin, chemerin, and resistin among the three groups (*p* < 0.01), but no significant difference was observed in the salivary levels of interleukin‐6 (*p* > 0.01). Analysis also revealed significant differences in appetite traits among the groups, with the highest appetite trait observed in pre‐diabetic subjects (*p* = 0.0002). The salivary level of chemerin was significantly associated with appetite traits regardless of diabetic status (*p* = 0.009). Appetite was also significantly related to BMI (*p* = 0.002) and waist circumference (*p* = 0.001) in all subjects. However, no significant relationship was observed between appetite and fasting plasma glucose or HbA1c levels (*p* > 0.01).

**Conclusion:**

The results of this study indicate that salivary levels of certain adipokines, such as leptin, chemerin and resistin, may be significantly higher in diabetic patients, although this is not true for all adipokines. While pre‐diabetic patients exhibited a higher level of appetite, no positive correlation was found between salivary adipokine levels (except chemerin) and appetite or anthropometric characteristics, irrespective of diabetic status.

## Graphical abstract

This study evaluates the relationship between salivary adipokines and obesity indicators in type 2 diabetes patients. The findings indicate differences among groups, with pre‐diabetics showing higher appetite; however, limited correlations exist between these adipokines and appetite or anthropometric features.

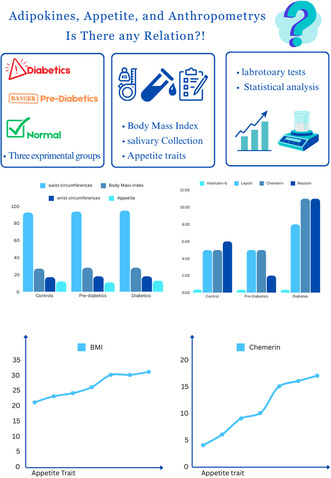

## 1 Introduction

Type 2 diabetes is one of the most serious diseases affecting modern humans, with its incidence rising in recent years [1]. Obesity has numerous detrimental consequences for health and is linked to a variety of disorders, including diabetes, cardiovascular diseases, high blood pressure, elevated blood lipid levels, certain types of cancer and increased mortality [2]. Individuals with obesity vary in terms of cardiovascular risk factors; often, the presence of these factors and insulin resistance is more significant than obesity itself [3]. Many studies have identified waist circumference (WC) as the most effective anthropometric marker for predicting the incidence of diabetes [4]. There is a strong correlation between type 2 diabetes and body mass index (BMI). Weight loss of 5%–10% in overweight patients can significantly reduce the risk of diabetes and improve glucose control, sometimes to the extent that insulin use can be reduced or discontinued. This degree of weight loss also leads to substantial improvements in blood lipid levels, blood pressure, and longevity [5].

White adipose tissue secretes various adipokines, including leptin, interleukin‐6, chemerin and resistin. Leptin, one of the most important adipokines, is synthesised and secreted by adipocytes. Circulating leptin levels are directly related to BMI or body fat. Leptin plays multiple roles, including appetite control, angiogenesis and insulin secretion. High levels of leptin are associated with an increased risk of type 2 diabetes, degenerative diseases, cardiovascular disease and autoimmune disorders. Conversely, decreased leptin levels can increase the risk of infections, particularly in malnourished individuals [6]. Interleukin‐6 regulates insulin sensitivity by downregulating lipoprotein lipase, triglyceride production and altering the expression of certain adipose tissue‐specific genes [7]. It enhances insulin secretion by activating intestinal L cells and pancreatic cells to produce and secrete glucagon‐like peptide‐1 (GLP‐1) [8]. Recently, chemerin has been identified as a novel lipid‐secreting factor crucial for adipocyte differentiation and insulin signalling [9]. Chemerin regulates immune responses, adipocyte differentiation, glucose and lipid metabolism, insulin sensitivity, insulin secretion and metabolic processes.

Resistin is a cysteine‐rich polypeptide that increases obesity and disrupts insulin action in mice [10]. In humans, resistin is also expressed in bone marrow cells, macrophages, pancreatic islets and other organs, leading to alterations in glucose metabolism, insulin resistance and pro‐inflammatory effects [11, 12]. Resistin is associated with obesity, periodontitis and cardiovascular disease [13].

Appetite traits in adulthood are relatively related to BMI. Hunot et al. demonstrated a correlation between high BMI and increased eating responsiveness, emotional overeating and enjoyment of food [14]. The higher incidence of diabetes in obese individuals, combined with the known effects of adipokines like leptin on appetite and insulin resistance [12], raises the question of whether there is a relationship between adipokines and appetite traits in adults.

Saliva is a hypotonic solution consisting of salivary acini, gingival crevicular fluid and oral mucosal secretions. Approximately 90% of saliva is secreted from the salivary glands, including the parotid, submandibular and sublingual glands. These highly permeable glands, surrounded by abundant capillaries and acini, facilitate the exchange of molecules, including adipokines secreted from body fat tissues. Therefore, circulating biomarkers, such as interleukin‐6 and leptin, show a good association between their levels in blood and saliva [15, 16].

Recently, saliva has been used as a clinical and diagnostic tool for both oral and systemic health, offering a quick and non‐invasive method that can reflect the levels of biomarkers found in blood [17]. Based on the evidence mentioned, this study aims to evaluate the relationship between the levels of different salivary adipokines—including leptin, chemerin, resistin and interleukin‐6—in patients with diabetes and anthropometric indices such as BMI, waist circumference, wrist circumference and appetite.

## 2 Methods

### 2.1 Study Population

This cross‐sectional study was conducted with referrals to the Hamadan Diabetes Clinic in 2023. All individuals who were referred to the clinic and met the inclusion criteria were included in the study. Participants were categorised into the following experimental groups based on their diabetes status: (1) diabetic patients [*N* = 35], (2) pre‐diabetic individuals [*N* = 35] and (3) control group [*N* = 30].

### 2.2 Ethics Statement

All participants were informed about the study conditions and assured that their decision to participate would not affect their treatment. Each participant provided informed consent before recruitment. The study design was approved by the Ethical Committee of the Vice Chancellery of Hamadan University of Medical Sciences, with the ethics code IR.UMSHA.REC.1401.740.

### 2.3 Sample Size

The sample size was determined using the formula provided by Taqdir et al., which estimated a correlation coefficient of 0.55 between waist circumference and leptin [18]. To reduce type I error to 1% and type II error to 10%, with a test power of 90% and confidence level of 99%, the sample size was calculated using the following formulas:

(1) $$n=\left[\left(z1-\alpha /2+z1-\beta \right)2+/\left(\mathrm{cr}\right)2\right]+3$$

(2) $$\mathrm{cr}=0.\text{5log}\left(\left(1+r\right)/\left(1-r\right)\right)$$

Therefore, the estimated appropriate sample size was 66 participants.

### 2.4 Inclusion Criteria

Diabetic participants were confirmed to have type 2 diabetes based on the following parameters:

- Fasting Plasma Glucose (FPG) ≥ 126 mg/dL (confirmed by two tests)
- HbA1c ≥ 6.5% (confirmed by two tests)
  Pre‐diabetic individuals had the following concentrations of FPG and HbA1c:
- 100 ≤ FPG < 126 mg/dL (confirmed by two tests)
- 5.7% ≤ HbA1c < 6.5% (confirmed by two tests)
  Subjects with FPG lower than 100 mg/dL and HbA1c lower than 5.7% were included in the control group.

### 2.5 Exclusion Criteria

Patients with the following conditions were excluded from the study: Immune system deficiencies, AIDS, Dry mouth, Sjögren's syndrome, Use of medications that reduce salivary secretion, History of salivary gland surgery, Head and neck radiotherapy, Cancer, Pregnancy.

### 2.6 Anthropometric Characteristics

For evaluating anthropometric characteristics, the following measurements were taken for all participants: Weight (kg), Height (m), BMI (kg/m^2^), Waist circumference (cm) and Wrist circumference (cm).

BMI was calculated as weight (kg) divided by the square of height (m^2^). Height was measured with the participant standing barefoot and their head in a horizontal position in the Frankfurt plane, using a tape measure placed 50 cm above the ground. Weight was measured with the subject wearing light clothing and standing on a digital scale placed on a hard surface. Measurements were recorded using a meter tape.

### 2.7 Appetite Trait

To evaluate appetite behaviour, participants completed the Adult Eating Behaviour Questionnaire developed by Hunot et al. [14] in the United Kingdom. The completed questionnaires were collected and analysed.

### 2.8 Salivary Analysis

Non‐stimulated saliva samples were collected from participants meeting the inclusion criteria using the spitting method. Participants were instructed to spit saliva every 60 s into a sterile 5 mL lidded microtube for 2–5 min. The collected samples were promptly transported to the laboratory, centrifuged at 2400 rpm for 15 min to remove contaminants, and then transferred to plastic tubes using a pipette. The samples were paraffinised and frozen at −80°C for subsequent evaluation. Levels of leptin, chemerin, interleukin‐6, and resistin were measured using human chemerin ELISA kit (ZellBio), human interleukin‐6 ELISA kit (ZellBio), human leptin ELISA kit (ZellBio) and human resistin ELISA kit (ZellBio, Tehran, Iran).

### 2.9 Method of Data Analysis

Data were gathered and analysed using SPSS version 26. The Kolmogorov–Smirnov test was used to assess the normal distribution of the data. One‐way ANOVA, Scheffe's test and Pearson's correlation coefficient tests were employed. The significance level was set at 0.01.

## 3 Results

There are 35 diabetics, 35 pre‐diabetics and 34 healthy participants in this study. Table 1 presents the demographic data of the participants according to their group. As shown, there were no significant differences between the groups regarding gender, age, smoking habits, or BMI (*p* > 0.01), except for a positive family history of diabetes. However, there were significant differences in fasting plasma glucose (FPG) and HbA1c levels among the experimental groups (*p* < 0.01).

**TABLE 1:** Characteristics of participants.

Variable	Control group	Pre‐diabetics group	Diabetics group	*p*‐Value
Male (%)	58.5	65.7	45.7	0.23
Age (years)	47.24 ± 13.78	50.91 ± 9.36	51.97 ± 11.7	0.22
History of smoking (per cent of smokers)	4 (11.76)	2 (5.71)	8 (5.71)	0.55
Family diabetes history (%)	13 (38.24)	9 (25.71)	23 (65.71)	0.003
FPG	76.91 ± 5.00	109.86 ± 8.02	170.31 ± 64.63	0.001
HbA1c	5.9 ± 0.3	5.72 ± 0.41	7.87 ± 1.61	0.001

*Note: p* = 0.01.

The mean and standard deviation of all measured variables, along with the *p*‐values from the one‐way ANOVA, are shown in Table 2.

**TABLE 2:** Mean and standard deviation of interleukin 6, leptin, chemerin and resistin.

Variable	Control group	Pre‐diabetics group	Diabetics group	*p*‐Value
Interlukin‐6	0.37 ± 0.13	0.34 ± 0.14	0.35 ± 0.13	0.63
Leptin	5.37 ± 1.76	5.25 ± 1.84	8.34 ± 2.38	< 0.001
Chemerin	5.27 ± 2.61	4.71 ± 2.22	10.99 ± 3.48	< 0.001
Resistin	5.80 ± 2.50	6.71 ± 2.04	10.72 ± 2.65	< 0.001
Appetite	12.10 ± 63	11.11 ± 81	13.11 ± 68	< 0.001
Body mass index (kg/m^2^)	26.98 ± 4.15	28.32 ± 4.15	27.88 ± 4.8	0.47
waist circumference (cm)	92.61 ± 9.52	93.71 ± 10.47	94.95 ± 8.68	0.34
Wrist circumference (cm)	17.1 ± 1.20	18.3 ± 2.1	18.2 ± 1.06	0.80

The results of the one‐way ANOVA test revealed that the concentration of salivary interleukin‐6 was not significantly different between the three experimental groups (*p* = 0.633). However, significant differences were observed in the salivary concentrations of leptin, chemerin and resistin (*p* < 0.001). Pairwise comparisons using Scheffe's test indicated that there were significant differences between the diabetic group and the other two groups (*p* < 0.001). No significant difference was found between the control group and the pre‐diabetic group.

One‐way ANOVA also showed a significant difference in eating behaviour among the groups (*p* < 0.001). Pairwise comparisons using Scheffe's test revealed that the control group had a significantly lower level of appetite compared to both the diabetic (*p* = 0.001) and pre‐diabetic groups (*p* = 0.002).

The differences in waist circumference (*p* = 0.343), wrist circumference (*p* = 0.078) and BMI (*p* = 0.47) among the groups were not significant (Table 2).

When comparing salivary adipokines (interleukin‐6, leptin, chemerin and resistin) to participants' appetite and anthropometric indices (body mass index, waist and wrist circumference), it was found that only the salivary level of chemerin had a significant relationship with appetite (*p* = 0.009) across all participants, regardless of their diabetic status. Pearson's correlation analysis also showed significant relationships between appetite and BMI (*p* = 0.002) and waist circumference (*p* = 0.001) among all subjects, including diabetic individuals, pre‐diabetic subjects and healthy controls. However, no significant relationship was observed between appetite levels and FPG and HbA1c indices.

## 4 Discussion

Obesity is characterised by excessive fat accumulation, with its severity and development influenced by adipokines. These molecules, which are secreted by adipose tissue, play critical roles in regulating adipocyte function, inflammation and insulin sensitivity. Adipokines are generally classified into two categories: “pro‐inflammatory” and “anti‐inflammatory.” Pro‐inflammatory adipokines exacerbate inflammation and insulin resistance, while anti‐inflammatory adipokines exert protective effects. An imbalance between these two types of adipokines can contribute to metabolic dysregulation and increased risk of conditions like type 2 diabetes [19].

Adipokines are involved in various biological processes, including adipogenesis, immune cell recruitment to adipose tissue and local (autocrine/paracrine) regulation of adipocytes. They also have endocrine effects, influencing multiple organs such as the brain, liver, pancreas, muscle, heart and vasculature [20].

Each adipokine operates through distinct biological pathways. Leptin, discovered in 1994, helps regulate appetite by increasing the synthesis of anorexigenic peptides and decreasing orexigenic peptides in the hypothalamus, thereby reducing hunger. Beyond appetite regulation, leptin is crucial for maintaining β‐cell mass and function [21]. It influences glucose homeostasis through two primary mechanisms: by binding to leptin receptors on pancreatic β‐cells, which inhibits insulin secretion [22], and by affecting β‐cell proliferation, apoptosis and insulin gene expression [23]. Chemerin, initially secreted as an inactive proprotein, can exert anti‐inflammatory effects on macrophages upon activation [24]. It plays a role in adipogenesis, adipocyte proliferation and the regulation of glucose and lipid metabolism [22]. In vitro studies suggest that chemerin enhances insulin‐stimulated glucose uptake in adipose tissue [9]. Resistin, produced by both adipocytes and adipose tissue macrophages, promotes oxidative stress and insulin receptor degradation, which impairs insulin signalling [25].

Given the rising prevalence of diabetes, numerous studies have explored the role of adipokines in metabolic disorders. In this study, we measured salivary levels of interleukin‐6, leptin, chemerin, and resistin in control, pre‐diabetic and diabetic groups. Attar et al. found that women with gestational diabetes had lower resistin levels compared to those without gestational diabetes and identified a significant relationship between postpartum metabolic syndrome and resistin and adiponectin levels during pregnancy [26]. Tvarijonaviciute et al. reported that salivary leptin levels in patients with type 2 diabetes were 1.2 times higher than in controls, aligning with our findings [27]. Okten et al. observed elevated salivary leptin levels in patients with gestational diabetes compared to healthy individuals, although this difference was not statistically significant [28].

In our study, we did not find significant correlations between adipokines and anthropometric indices across the groups. However, a notable relationship was observed between salivary chemerin levels and appetite. Despite this, there were no significant differences in body mass index (BMI), waist circumference or wrist circumference among the groups, suggesting that BMI alone may not fully capture the metabolic differences between diabetic, pre‐diabetic, and control groups [29]. The average BMI for the diabetic group was 27.78, compared to 26.94 in the control group, indicating that other factors such as genetics, diet and geographic location might also influence anthropometric measures.

Participants completed a translated questionnaire to assess appetite and nutritional status. Analysis using one‐way ANOVA revealed a significant difference in appetite among the groups, with diabetic and pre‐diabetic participants exhibiting higher appetite levels compared to controls. Yilmaz et al. reported a higher prevalence of binge eating disorder among overweight individuals compared to those who are normal weight or underweight—26.7%, 23.8% and 21.6%, respectively [30]. Our findings support this, suggesting that individuals with larger anthropometric measurements tend to overeat more, and this relationship can be cyclical and reinforcing.

Our study has several limitations, including a small sample size, potential uncontrolled confounders and its cross‐sectional design, which limits causal interpretations. Future research should focus on the functional characterisation of adipokines and their clinical implications. Longitudinal studies that track changes in salivary adipokine levels over time and intervention studies examining the impact of lifestyle modifications on adipokine profiles are highly recommended.

## 5 Conclusion

According to the results of this study, the salivary levels of certain adipokines, such as leptin, chemerin and resistin, can be significantly higher in diabetic patients, though this is not the case for all adipokines. While pre‐diabetic patients exhibited a higher level of appetite, no positive correlation was found between salivary adipokine levels (except chemerin) and appetite or anthropometric characteristics in the population, regardless of diabetic status.

## Author Contributions

All authors are jointly designed the study, developed the methodology, collected the data, performed the analysis and wrote the manuscript.

## Conflicts of Interest

The authors declare no conflicts of interest.

## Data Availability

The data that support the findings of this study are available from the corresponding author upon reasonable request.
